# Supplementary material for: Reliable monitoring of respiratory function with home spirometry in people living with amyotrophic lateral sclerosis
Source: Front Neurol. 2025 Jun 24;16:1588992. doi: 10.3389/fneur.2025.1588992 (PMC12235912; doi:10.3389/fneur.2025.1588992)
Supplement: Supplementary file 1 [file Presentation_1.pdf]

Reliable Monitoring of Respiratory Function with Home Spirometry in People Living with Amyotrophic Lateral Sclerosis

Supplementary Materials

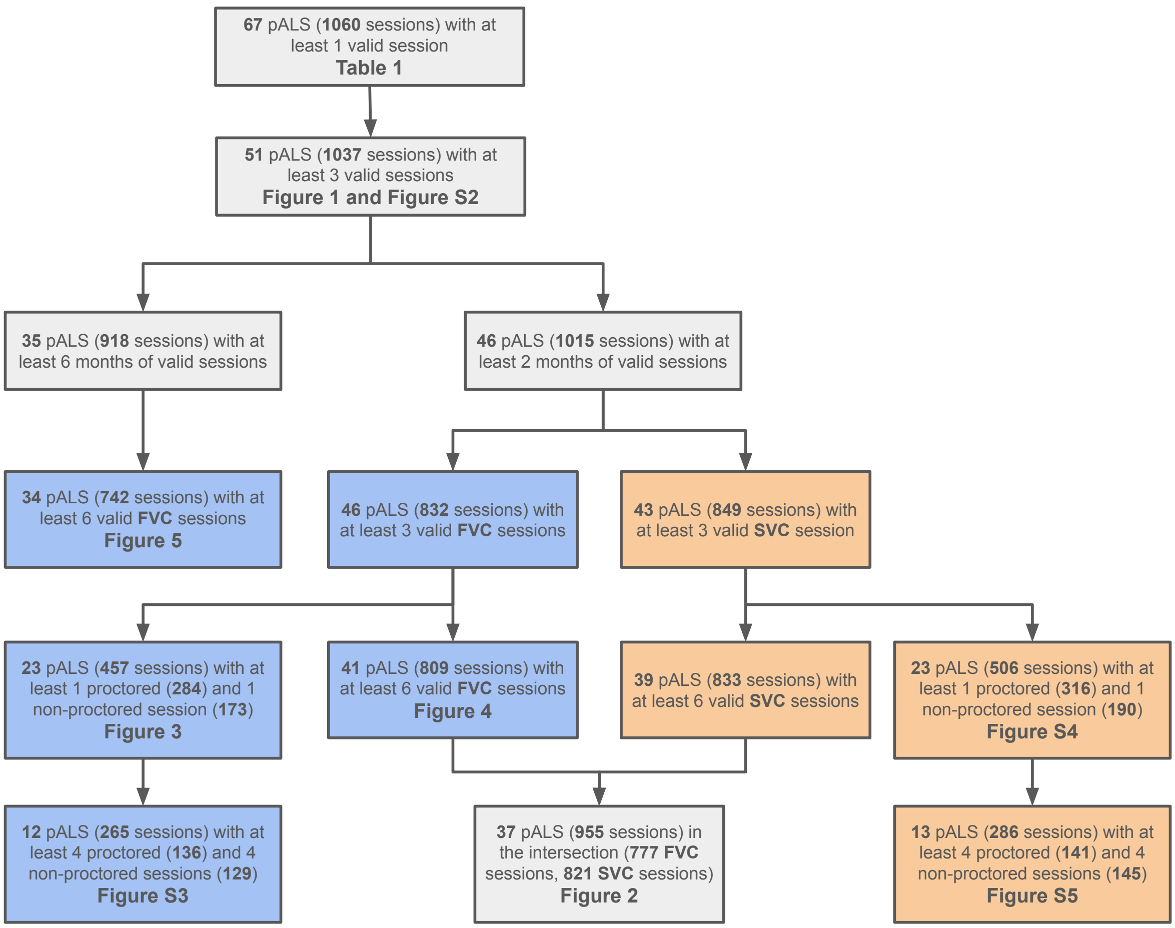

Figure S1: Overview of the data flow across the figures presented in this work. Blue and orange boxes correspond FVC and SVC data respectively.

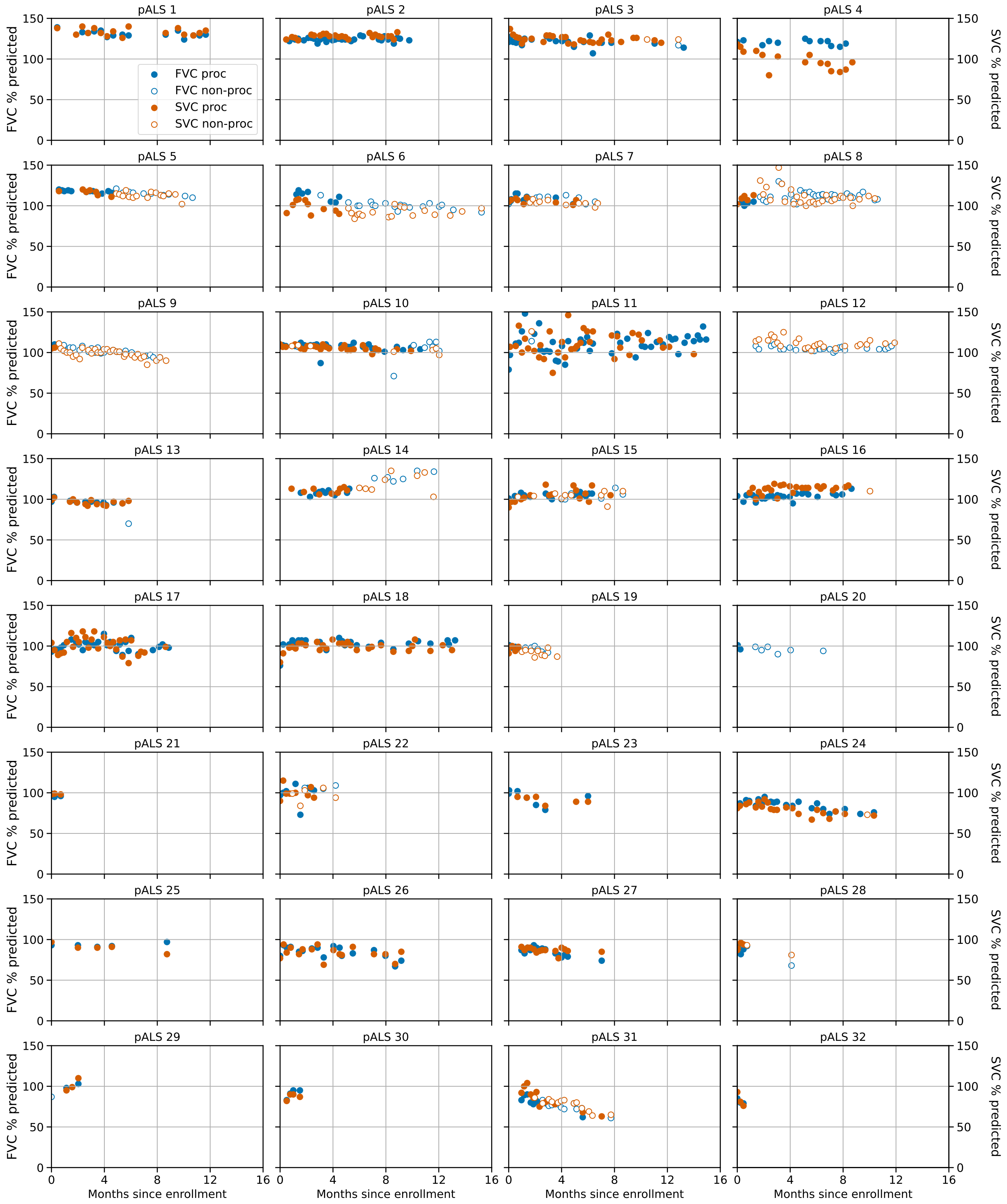

Figure S2: **Evolution of SVC and FVC for pALS in the Radcliff Study.** Trajectories of FVC are shown in blue and SVC in brown for the set of 51 pALS that completed at least 3 sessions (filled points are proctored, hollow non-proctored).

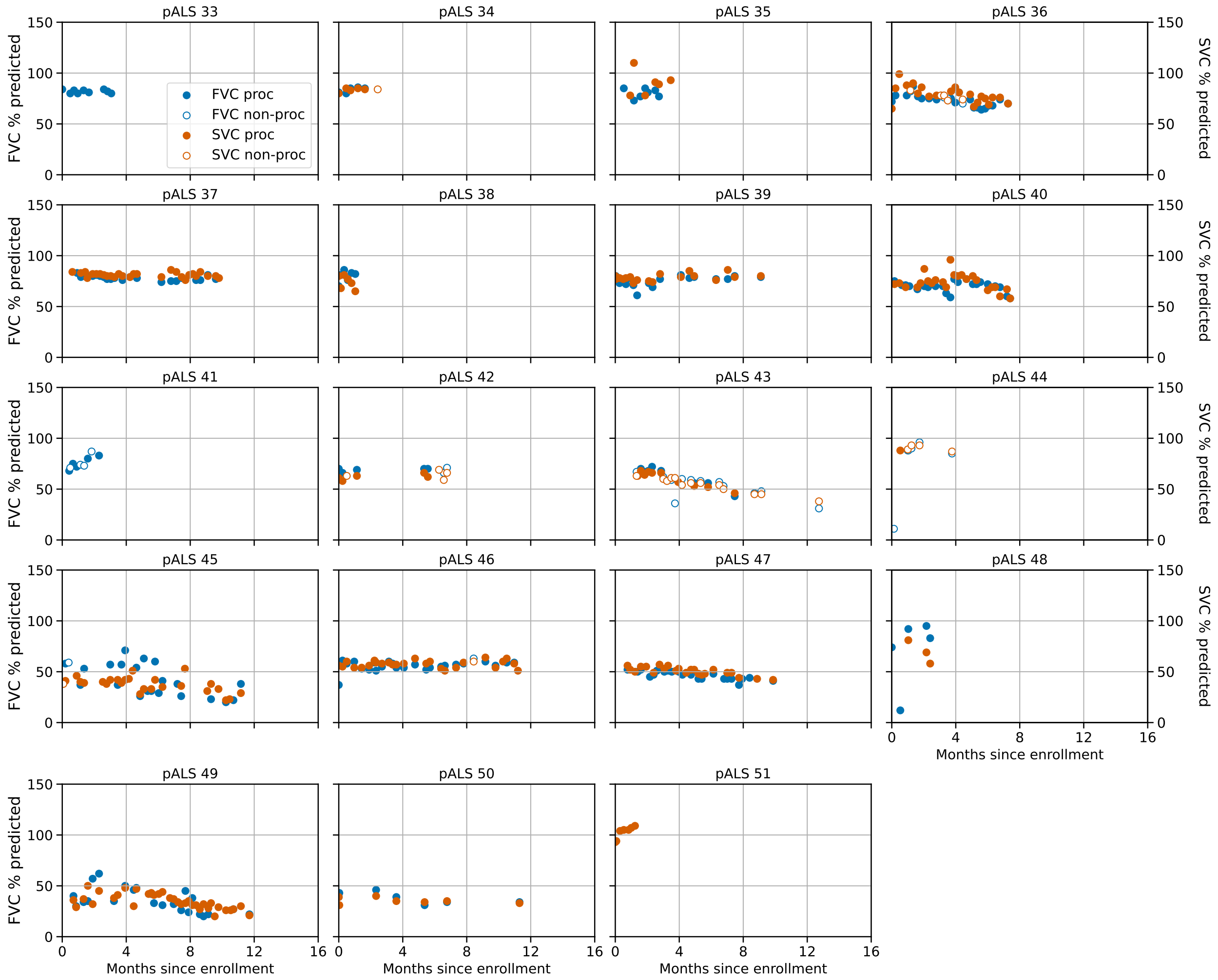

Figure S2: **(cont.) Evolution of SVC and FVC for pALS in the Radcliff Study.** Trajectories of FVC are shown in blue and SVC in brown for the set of 51 pALS that completed at least 3 sessions (filled points are proctored, hollow non-proctored).

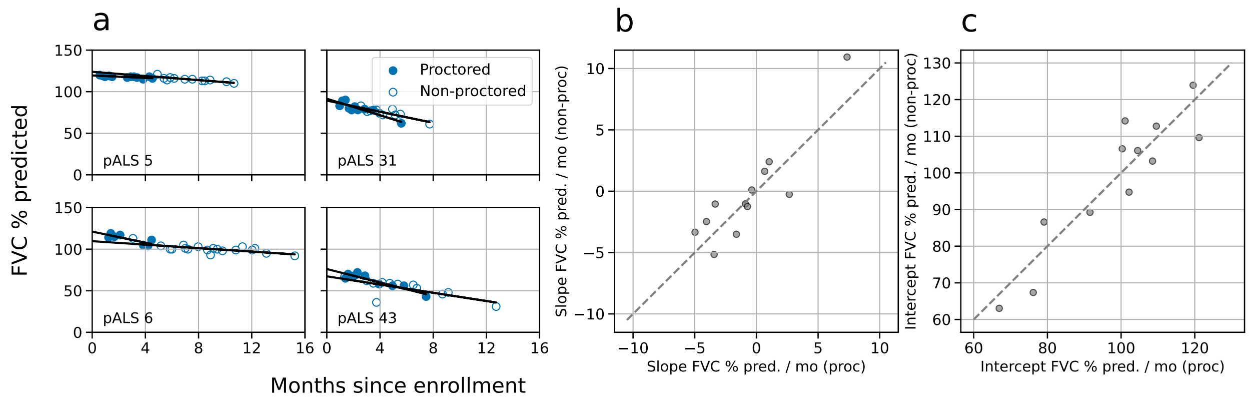

Figure S3: **Individual regressions for proctored and non-proctored FVC data.** (a) FVC time series examples for pALS 5, 6, 31 and 43 (See Figure S2) with linear regressions for proctored (blue) and non-proctored (pale blue) data after outlier removal. (b) Comparison of slopes and (c) intercepts for the 12 pALS in the Radcliff Study with at least 4 proctored and 4 non-proctored FVC sessions in the dataset (265 sessions). Good Spearman correlations for both slopes (ρ=0.853, p<0.001) and intercepts (ρ=0.797, p=0.002) show consistency across time for both conditions.

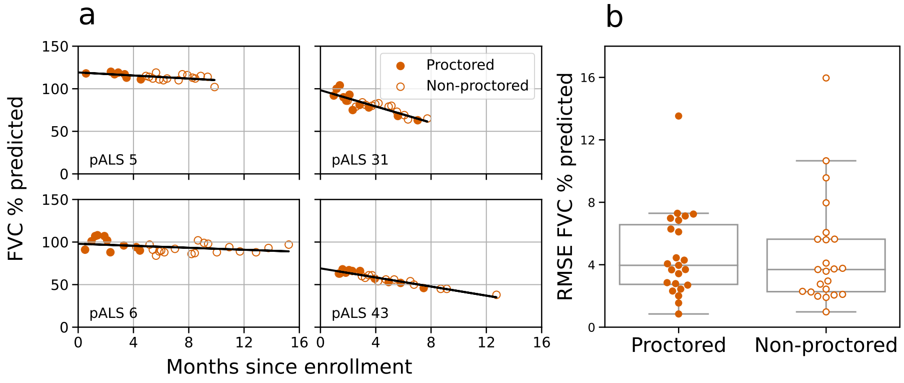

Figure S4: **Consistency between proctored and non-proctored SVCs.** (a) Time evolution of SVC for pALS 5, 6, 31 and 43 (see Figure S2) with orange points representing proctored sessions and white points representing non-proctored sessions. (b) Linear regressions were computed for each pALS with at least 3 SVC sessions, at least 1 proctored and 1 non-proctored in the dataset and at least 2 months of valid sessions (23 pALS, 506 sessions with 316 proctored and 190 non-proctored), showing no statistical difference in the RMSE between the two cohorts (T-statistic = −0.11, $p > 0.9$, $N = 23$).

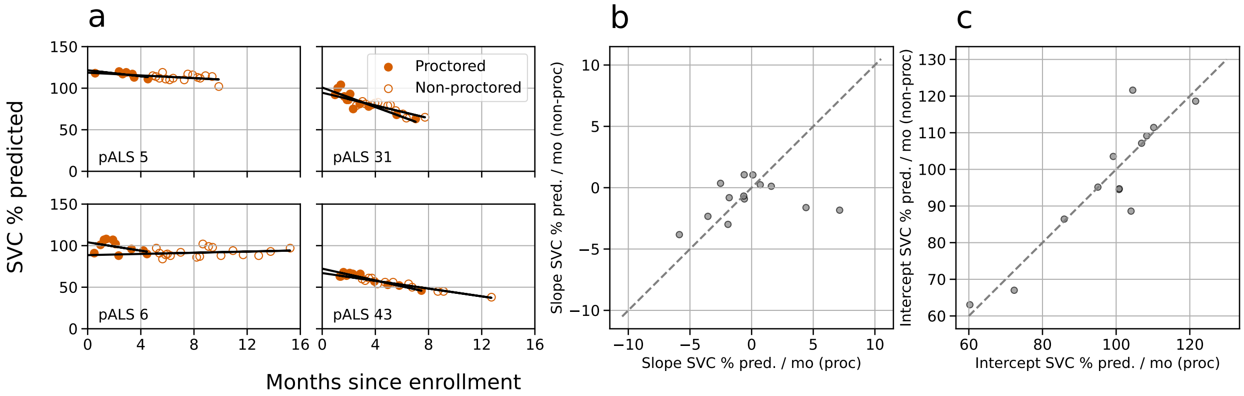

Figure S5: **Individual regressions for proctored and non-proctored SVC data.** (a) SVC time series examples for pALS 5, 6, 31 and 43 (see Figure S2) with linear regressions for proctored (dark) and non-proctored (light) data after outlier removal. (b) Comparison of slopes and (c) intercepts for the 13 pALS in the Radcliff Study with at least 4 proctored and 4 non-proctored SVC sessions in the dataset (286 sessions). Good Spearman correlations for both slopes (ρ=0.286, p=0.344) and intercepts (ρ=0.846, p<0.001) show consistency across time for both conditions.
